# RNA-Seq Reveals Infection-Related Gene Expression Changes in *Phytophthora capsici*

**DOI:** 10.1371/journal.pone.0074588

**Published:** 2013-09-03

**Authors:** Xiao-Ren Chen, Yu-Ping Xing, Yan-Peng Li, Yun-Hui Tong, Jing-You Xu

**Affiliations:** College of Horticulture and Plant Protection, Yangzhou University, Yangzhou, China; Auburn University, United States of America

## Abstract

*Phytophthora capsici* is a soilborne plant pathogen capable of infecting a wide range of plants, including many solanaceous crops. However, genetic resistance and fungicides often fail to manage *P. capsici* due to limited knowledge on the molecular biology and basis of *P. capsici* pathogenicity. To begin to rectify this situation, Illumina RNA-Seq was used to perform massively parallel sequencing of three cDNA samples derived from *P. capsici* mycelia (MY), zoospores (ZO) and germinating cysts with germ tubes (GC). Over 11 million reads were generated for each cDNA library analyzed. After read mapping to the gene models of *P. capsici* reference genome, 13,901, 14,633 and 14,695 putative genes were identified from the reads of the MY, ZO and GC libraries, respectively. Comparative analysis between two of samples showed major differences between the expressed gene content of MY, ZO and GC stages. A large number of genes associated with specific stages and pathogenicity were identified, including 98 predicted effector genes. The transcriptional levels of 19 effector genes during the developmental and host infection stages of *P. capsici* were validated by RT-PCR. Ectopic expression in *Nicotiana benthamiana* showed that *P. capsici* RXLR and Crinkler effectors can suppress host cell death triggered by diverse elicitors including *P. capsici* elicitin and NLP effectors. This study provides a first look at the transcriptome and effector arsenal of *P. capsici* during the important pre-infection stages.

## Introduction

It has been found that more than 80 species of the genus *Phytophthora* can cause tens of billions of dollars of damage each year to a wide variety of agriculturally and ornamentally important crops worldwide [1], [2]. They also cause devastating plant diseases in forests, leading to severe losses [3]. These filamentous pathogens belong to the oomycetes, a class of eukaryotes that is phylogenetically related to heterokont brown algae instead of true fungi [4].

*Phytophthora* species are regarded as hemibiotrophs since for part of their life cycle they maintain a biotrophic relationship with their host. A series of cell types are formed prior to host cell penetration, including sporangia, zoospores, cysts, and germinating cysts with germ tubes, all of which are important for plant infection and disease development [5]. The infection by root pathogenic oomycetes is initiated by the release from sporangia of motile, biflagellate and wall-less zoospores, which encyst and adhere to the host surface following a chemotactically and electrotactically swimming stage. The cysts germinate by forming the germ tubes and start to penetrate the plant cuticle directly with the aid of secreted enzymes and colonize host tissues. Previous studies have shown that these pre-infection structures are rich in molecules involved in establishment of infection and elicitation of plant defenses [5]–[9]. The unraveling of the molecular processes regulating the life cycle of *Phytophthora* is therefore important to identify determinants of pathogenesis and develop appropriate control strategies.

The heterothallic oomycete *Phytophthora capsici* Leonian can cause seed, root, crown, foliar and fruit rot on a number of important crops, such as solanaceous crops (pepper, eggplant, tomato), cucurbits (cantaloupe, cucumber, squash, pumpkin, watermelon) and bean crops [1], [10], [11]. *P. capsici* is also recorded as a pathogen of *Allium cepa*, *Nicotiana benthamiana* and *Arabidopsis thaliana* [12]–[15]. To date, the diseases caused by *P. capsici* have become devastating ones of global economic importance. The cost that *P. capsici* inflicts upon worldwide vegetable production is estimated to be $1 billion every year [14]. Unfortunately, the control of *P. capsici* is a difficult task as there has been no available effective chemical or cultural strategy [14]. In order to improve methods for controlling *Phytophthora* diseases, it is essential to understand the molecular mechanisms by which the pathogen suppresses or escapes the host plant defenses.

Recent studies on plant pathogenic oomycetes have demonstrated that these pathogens accomplish their penetration and colonization of host plants by manipulating their hosts through a diverse arsenal of secreted proteins (effectors) [16]–[21]. According to their potential targeting sites in the host plant, these secreted effector proteins are classified into two classes, apoplastic and cytoplasmic effectors [16]. Apoplastic effectors, including elicitins, PcF/SCR-like proteins and NLPs (NPP1-like proteins), are located at the interface between pathogen and its host and fulfill a function on the outside of the host cell. Elicitins, one type of pathogen-associated molecular patterns (PAMPs), can trigger plant cell death response, normally known as hypersensitive reaction (HR) that is characteristic of plant defense response. These proteins share a conserved 98-amino-acid domain with a characteristic signature of six cysteine residues that form three distinct disulfide bonds [16]. Members of the PcF/SCR toxin family, small cysteine-rich proteins, are thought to be involved in the induction of plant cell death [16], [22]. Other toxins secreted by oomycetes belong to NLPs that elicit plant cell death in dicotyledonous plants [23], [24]. In contrast, cytoplasmic effectors are able to translocate inside host cells where they interfere with the host defense responses. Two important groups of translocated effectors are RXLR and CRN (Crinkler) protein families [16]. RXLR effectors are named after an N-terminal RXLR amino acid motif contained by the first characterized effectors of this class, where ‘X’ denotes a nonconserved amino acid residue [25], [26]. This motif assists in the translocation of the proteins into the host’s cytoplasm where the effectors function as virulence or avirulence factors depending on the host genotype. Host translocation may also occur with variations of the RXLR motif (such as QXLR, GXLR) or even in the absence of any such motif may [27]–[31]. A structural dissection of these proteins has revealed a signal peptide followed by an RXLR motif at the N-terminus, which are required for secretion and targeting of the proteins whereas the C-terminus executes the actual effector activity [27], [32], [33]. A second, well-studied class of host-translocated effectors is the Crinklers (CRNs), named for their ability to trigger host leaf-crinkling and necrosis accompanied by an induction of host defenses [34]. Similar to the RXLR effectors, the Crinklers are modular proteins with a signal peptide, conserved N-termini and highly diverse C-terminal domains [18]. The conserved Crinkler N-termini, harboring a distinct LXLFLAK motif and the conserved DWL domain, are functionally equivalent to the N termini of RXLR effectors and required for effector targeting and host translocation [18], [35], [36]. Deletion and expression analyses in *N. benthamiana* have defined important C-terminal domains that are required for induction of cell death [18]. Stable transformation-based gene silencing experiments demonstrated that two *Phytophthora sojae* CRNs are required for successful infection of soybean [37]. van Damme et al. [38] reported that CRN8, a host-translocated CRN effector of *Phytophthora infestans* is a functional RD kinase inside host cell and that heterologous expression of CRN8 *in planta* resulted in enhanced virulence by *P. infestans*.

Although the impact of crop loss caused by *P. capsici* has increased in recent years, little is known about the molecular basis of the pathogenicity of *P. capsici*. To explore recently-released *P. capsici* reference genome sequence and to accelerate research aiming to address the molecular mechanisms underlying host plant infection, defining the *P. capsici* transcriptome therefore is an important strategy to interpret the functional elements of the genome and to dissect the molecular events that accompany pathogenesis. Gene transcripts can be profiled by a number of techniques aimed at isolating differentially expressed genes (DEGs), such as serial analysis of gene expression [39], microarray [40], [41], and cDNA libraries [42]. During the past few years, however, next-generation sequencing (NGS) technology has been an efficient route for generating enormous sequence collections that represent expressed genes and quantitate expression levels in particular organs, tissues, or cells, under different treatments or conditions [43], [44]. In this study, we employed the *P. capsici* draft genome as a reference for a transcriptional study using Illumina RNA-Seq [43]. RNA samples from three important life cycle stages were deeply sequenced. *P. capsici* mycelia growing in culture medium (hereafter referred to as MY), zoospores (hereafter referred to as ZO) and germinating cysts with germ tubes (hereafter referred to as GC) were analyzed, and transcript abundances compared to characterize gene expression patterns during these pre-infection stages. We specifically focus on the identification of RXLRs, CRNs, NLPs and elicitins, predicting that they would be more highly expressed in pre-infection or infection stages versus vegetative growth stage. Our results supported our prediction, revealing more transcriptional changes in *P. capsici* during the host infection stages. Ectopic expression assays in *N. benthamiana* showed that *P. capsici* NLP and elicitin effectors can induce cell death whereas RXLR and CRN effectors can suppress host cell death triggered by them but also other different elicitors. This study provides a critical step to characterize the mechanisms of pathogenicity and virulence of *P. capsici*.

## Materials and Methods

### Oomycete materials

**Figure 1:**
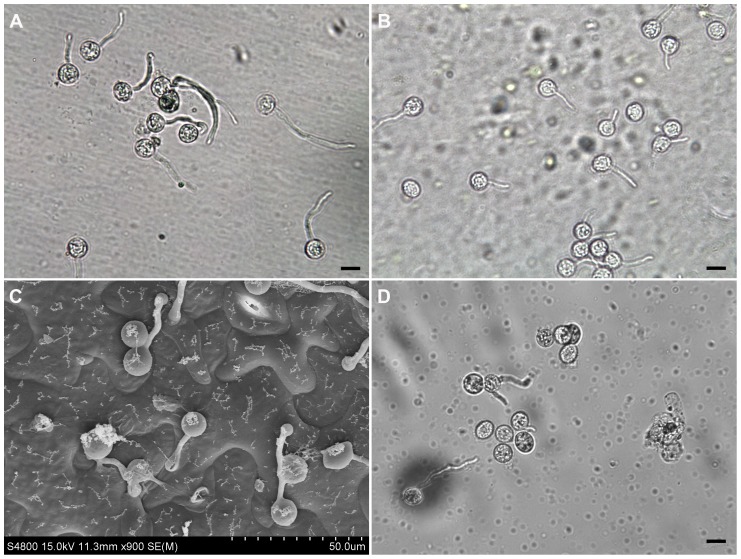
The *Phytophthora capsici* cysts germinating on different surfaces. The cysts were germinated on a cellophane membrane that was placed on the top of a *Nicotiana benthamiana* leaf (A), or directly on *N. benthamiana* leaves (B-C), or directly in water (D). Photos were taken at 70 (A), 60 (B-C) and 90 (D) min post-inoculation. To make samples for observation in (B), free water around the inoculation sites was absorbed away by filter paper and germinating cysts with some leaf tissue were peeled off using the sticky tape method [79]. The cysts germinating directly on *N. benthamiana* leaves were also observed using Cryoscanning electron microscopy (Hitachi S-4800 SEM) according to the instruction manual (C). The germinating cysts (A, B and D) were observed under an Olympus microscope. Scale bar  =  10 µm.

Strain Pc537 of *P. capsici* was isolated from diseased pepper in China, preserved in liquid nitrogen and routinely maintained on 10% V8 agar media at 25 °C in the dark [1]. Samples of mycelia, sporangia, zoospores and germinating cysts were prepared as the following steps. Hyphal tip plugs were used to inoculate 30 mL of sterile clarified 10% V8 broth in 90-mm Petri dishes. Stationary mycelial cultures were incubated at 25 °C in the darkness for 4 days. To induce the sporangium formation, the agar culture from each 1 to 2-week-old Petri dish was cut into six pieces, covered with sterile distilled water and kept in Petri dishes at 25 °C in darkness. Then, the water in dishes was replaced by new sterile distilled water every 12 h. After 3 to 4 times changes of distilled water, plenty of sporangia were produced. To encourage the release of zoospores, the culture with sporangia was placed at 4 °C for 30 min, and then incubated at room temperature for another 30 min. The sporangia and zoospores were checked under an Olympus light microscope. Harvesting of sporangia and zoospores were performed according to Randall et al. [45]. Cysts were obtained by rigorously vortexing the zoospore suspension for 30 s. After this step, a solution containing 20,000 encysted zoospores was dropped onto a cellophane membrane that was pre-treated and placed on a detached leaf of *N. benthamiana* following the previously described procedure [46]. This was established to mimic the cyst germinating on host surface but to avoid the inclusion of plant biomass into further analysis. A moist Whatman filter paper was already placed underneath the leaf to keep humidity in a sterile Petri dish. Incubation was conducted at 25 °C in the darkness. The cyst germination was regularly observed every 10 min under the microscope after 40 min post-inoculation. Germinating cysts were collected when the average length of germ tubes of 80% germinating cysts was twice the length of the average diameter of cysts (Figure 1A). Sporangia, zoospores or germinating cysts were collected by centrifugation at 1,500 *g* for 10 min and immediately preserved in liquid nitrogen for RNA isolation.

### Plant growth and inoculation

*N. benthamiana* plants were grown in Styrofoam cups containing sterile soil and placed in greenhouse at 25 °C with a cycle of 16 h day and 8 h night, until 5 to 6 weeks old.

The plants were watered two to three hours prior to inoculation to insure that the soil was wet. Each tobacco plant was inoculated by directly decanting 9 mL of freshly prepared zoospore suspension (1×10^5^ zoospores·mL^−1^) around the rhizosphere. The plants inoculated with sterile water served as controls. All the plants were moved into a growth chamber with the setting to a temperature of 25±1°C, a relative humidity of 98% ± 1% and a cycle of 16 h day and 8 h night. No evident macroscopic symptoms were observed in the roots until 36 h post-inoculation (hpi) (Figure S1); therefore, we cut the portion of the roots that had been in direct contact with the inocula for the earlier time points. The infected portions of the inoculated roots were harvested at 1.5, 3, 6, 12, 24, 36, 72 hpi (Figure S1). Additional plants, inoculated and mock-inoculated, kept for 5 days after inoculation were totally dead or not infected at all, respectively. This was used to ensure the success of the inoculations. All collected samples were flash frozen in liquid nitrogen and stored at –75°C until RNA extraction.

### Isolation of nucleic acids

Genomic DNA of *P. capsici* isolate Pc537 was extracted from mycelia using CTAB (cetyltrimethylammonium bromide) method [47]. RNA was extracted from samples using RNAiso Plus reagent (Takara Biotechnology (Dalian), China) according to the manufacturer’s instructions. The quality and quantity of both DNA and RNA were checked by gel electrophoresis and spectrophotometrically. Prior to cDNA synthesis, RNA samples were treated with DNase I (TaKaRa). RT-PCR (without reverse transcriptase) running 45 cycles was used to check that RNA samples were not contaminated with genomic DNA. After that, all RNA samples were stored at –75°C until further use.

### Library preparation and sequencing

Total RNA was isolated from each of three samples (MY, ZO and GC). Total RNA of each sample was processed for Illumina RNA-Seq. The RNA-Seq libraries were constructed according to the manufacturer’s instructions of Illumina Gene Expression Preparation Kit (Illumina, San Diego, CA, USA). Briefly, poly (A+) mRNA was isolated from total RNA using Magnetic Oligo(dT) Beads (Illumina). To avoid priming bias, mRNA was fragmented by the RNA fragmentation kit (Ambion, Austin, TX, USA) before cDNA synthesis. First-strand cDNA was synthesized from the cleaved RNA using SuperScript III reverse transcriptase and random hexamer primers (Invitrogen, Carlsbad, CA, USA), followed by second-strand cDNA synthesis using DNA polymerase I (New England BioLabs, Ipswich, MA, USA) (NEB). Residual RNA in the reaction was removed by using RNase H (Invitrogen). After purification by QIAquick PCR Purification Kit (QIAGEN, Dusseldorf, Germany), the double-strand cDNA was treated to repair ends by T4 DNA polymerase, Klenow Enzyme, and T4 polynucleotide kinase (NEB). A single ‘A’ base was then added to the 3′ end of cDNA by Klenow 3′ to 5′ exo-polymerase (NEB) for DNA ligation to PE Adapter Oligo Mix (Illumina) which had a single ‘T’ base overhang. After ligation, the DNA was purified by MinElute PCR Purification Kit (QIAGEN) and separated by electrophoresis on a 2% agarose gel. The range of cDNA fragments (200±25 bp) were excised from the gel and purified for downstream analysis. PCR amplification with 10 to 12 cycles using Phusion polymerase (Finnzymes, Espoo, Finland) was performed with primers complementary to the adapter sequences to enrich the samples for the desired fragments. The PCR products were purified using QIAquick PCR Purification Kit and dissolved in 30 µL of QIAGEN EB buffer. These DNA fragments represented the designated mRNA library. Subsequently, the purified DNA fragments were single-end sequenced on the Illumina Cluster Station and Illumina Genome Analyzer IIx sequencing platform. Image recognition and base calling were performed using the Illumina Pipeline.

### Sequence bioinformatics and differential expression analysis

The raw reads were first cleaned by eliminating adapters and low-quality sequences (reads with ambiguous bases ‘N’), and reads with more than 10% Q <20 bases (those with a base quality less than 20). The minimum acceptance threshold in length was 60 bp because small reads might represent sequencing artifacts.

Several complementary approaches were utilized to annotate the sequences. The read sequences were initially annotated based on the *P. capsici* reference genome (LT1534 v11.0) (http://genome.jgi-psf.org/Phyca11/Phyca11.home.html). Searches were conducted using the BLAST-Like Alignment Tool (BLAT) [48] with all default parameters but -tileSize = 9 and -stepSize = 6. Gene names were assigned to the sequences based on the best hit (highest score). Each matched gene model sequence was then downloaded from the *P. capsici* genome database and used for the following comparisons. Due to the availability of the well-annotated *P. infestans* genome, comparison of expressed *P. capsici* genes was carried out against this genome database (http://www.broadinstitute.org/annotation/genome/phytophthora_infestans/MultiHome.html) using BLASTn (*E*-value cutoff of 1e-10) [49]. The *P. capsici* genes were also compared against the NCBI Nt database (BLASTn), Swiss-Prot, NCBI Nr, TrEMBL databases (BLASTx) with an *E*-value of 1e-5 [49]. Functional annotation by gene ontology terms (GO, http://www.geneontology.org) was analyzed by Blast2GO software (BLASTx, *E*-value cutoff of 1e-5). The sequences were also aligned to the Clusters of Orthologous Groups (COG) database (http://www.ncbi.nlm.nih.gov/COG/) to predict and classify functions (BLASTx, *E*-value cutoff of 1e-5). The Kyoto Encyclopedia of Genes and Genomes (KEGG) pathways were assigned to the sequences using the online KEGG Automatic Annotation Server (KAAS) (http://www.genome.jp/kegg/kaas/) (BLASTx, *E*-value cutoff of 1e-5). The bi-directional best hit (BBH) method was used to obtain KEGG Orthology (KO) assignment [50]. The output of KEGG analysis includes KO assignments and KEGG pathways that are populated with the KO assignments.

To compare the gene expression level in different libraries, the transcript level of each expressed gene was calculated and normalized to the reads per kilobase of exon model per million mapped reads (RPKM) [51]. The RPKM measure of read density reflects the molar concentration of a transcript in the starting sample by normalizing for RNA length and for the total read number in the measurement. Significance of differential gene expression was determined by using General Chi-squared test integrated in IDEG6 software (http://telethon.bio.unipd.it/bioinfo/IDEG6/) [52]. IDEG6 is a web tool designed to identify differentially expressed genes in multiple tag experiments and assigns a *P*-value by statistical analysis of matrices of expression data. An expression profile matrix was built representing the frequency of unique genes in libraries using RPKM integer values, and imported into the IDEG6 software to conduct General Chi-squared test. *P* values from this method were adjusted to account for multiple testing by use of the false discovery rate (FDR). Assume that we have picked out R DEGs in which Y genes really show differential expression and the other V genes are false positive. If we decide that the error ratio ‘‘Q =  V/R’’ must stay below a cutoff (e.g. 5%), we should preset the FDR to a number no larger than 0.05 [53]. FDR was applied to determine the threshold of *P* value in analyses. Here, an FDR-adjusted *P* value < 0.01 was considered a statistically significant result. Genes with an adjusted *P* value < 0.01 and the absolute value of log_2_ (expression fold change) ≥ 1 were deemed to be differentially expressed.

### RT-PCR analysis

RNA was reverse transcribed into cDNA using M-MLV Reverse Transcriptase (RNase H Minus) and random hexamer primers (TaKaRa) following the manufacturer’s protocol.

The primers used in the reactions are listed in Table S1. PCR reactions took place on a thermo cycler LS-96G (Thermofisher, Dubuque, IA, USA) with the following programs: 94 °C for 5 min, followed by 29 cycles of 95 °C for 15 s, 46 to 60 °C for 30 s, and 72 °C for 1 min, and a final extension of 72 °C for 2 min. The housekeeping gene encoding ubiquitin-conjugating enzyme (*Ubc*) (the DOE Joint Genome Institute [JGI] protein ID 510705) was used as the internal control [54]. Genomic DNA of *P. capsici* was used as a control template to ensure that PCR reactions worked properly while water control was included to minimize the misinterpretation of the results. When gene expression was monitored in infected *N. benthamiana*, cDNA from mock-inoculated plants was used as an additional negative control for each primer pair. This was to exclude the possibility that the amplification was due to homologous plant genes. All RT-PCRs were performed at least three times.

### Gene cloning and construct development

To amplify the full-length genes, the gene models of *P. capsici* reference genome were used to design primers (Table S1). The genes were initially PCR amplified from *P. capsici* Pc537 genomic DNA and cloned into a TA vector pMD-18T (TaKaRa). Signal peptide scores for each gene model were predicted by the SignalP v3.0 server [55].

To construct the transient assay vectors, gene fragments were PCR amplified using high-fidelity DNA polymerase (PrimeSTAR® HS DNA Polymerase; TaKaRa) and individually sub-cloned directly into a binary potato virus X (PVX) vector pGR107 [56] predigested by restrict enzyme *Sma*I (NEB). Primers (Table S1) towards elicitin and NLP genes were designed to amplify the full-length gene sequences including signal peptide-encoding region from cDNA samples in which the genes were expressed. In contrast, the PVX::*RXLR* or PVX::*CRN* constructs were designed to express RXLR or CRN without the predicted N-terminal signal peptide. Their gene fragments were amplified directly from the genomic DNA since both categories don’t contain any introns. The sequencing was done using PVX-F/PVX-R primers [34] complementary to the vector sequence and flanking the insert to identify the insert orientation and PCR-derived errors.

### Multiple sequence alignment of *P. capsici*

Sequence alignment was performed to investigate the relationship of the predicted effectors with other *Phytophthora* spp. using the publicly available program BioEdit (v7.2.0) [57]. Initially, five TA clones for each gene were sequenced by DNA Sanger sequencing using PVX primers. For genes larger than 1 kbp in length, additional gene-specific primers were designed at an interval of 700 bp. The full-length sequence, translated using BioEdit, was used to perform a protein–protein BLAST (BLASTP) search in FungiDB database (http://fungidb.org; v2.3) using default parameters, and the protein sequences of other *Phytophthora* species were downloaded from the database. These sequences were then employed for multiple sequence alignments using BioEdit.

### *Agrobacterium tumefaciens* infiltration assay

The binary construct was introduced independently into *A. tumefaciens* strain GV3101 by electroporation and transformants were selected with rifampicin (25 µg·mL^−1^) and kanamycin (50 µg·mL^−1^). After growing at 28°C in Luria-Bertani (LB) agar plates supplemented with antibiotics for 2 days, individual colonies were extracted with NaOH (2 mM) lysis solution and PCR amplified to verify that the correct clones were selected for plant transient expression assay.

For infiltration, the recombinant strains were grown in LB broth plus appropriate antibiotics at 28 °C and 200 rpm for 2 days. The cells were collected by centrifugation at 3,000 *g* for 5 min, washed three times in 10 mM MgCl_2_, and resuspended in 10 mM MgCl_2_ to an optical density at 600 nm of 0.4 to 0.6, and then incubated at room temperature for 1 to 3 h prior to infiltration. Infiltration experiments were conducted on 5- to 6-week-old *N. benthamiana* plants. For cell death induction experiments, small nicks were made in each leaf with a needle and then 20 to 40 µL of cell suspension carrying the respective constructs was infiltrated through each nick using a blunt syringe. For cell death suppression experiments, *A. tumefaciens* cells carrying either of RXLR and CRN genes were infiltrated initially following the above method. Then, 24 h later the same infiltration site was challenged with *A. tumefaciens* cells carrying the cell death-inducing genes: *Bax* (triggering HR-mimicking cell death in plants) [58], a *P. sojae* CRN effector gene *PsCRN63* [37], a *P. sojae* RXLR effector gene *Avh241* [59], [60], *PsojNIP* [61], *P. infestans INF1* [32], a combination of *P. infestans Avr3a* and potato *R3a* as an HR inducer [62], or the effector genes characterized in this study. *A. tumefaciens* strains carrying *GFP* gene alone were infiltrated in parallel as negative controls. Each assay consisted of at least four plants inoculated on three leaves (total of twelve leaves). Symptom development was monitored visually 3 to 10 d after infiltration, and photographs were taken at 5 d. All of the experiments were repeated at least three times.

## Results

### Gene expression profiling using Illumina sequencing

To understand the molecular bases of pathogenicity of *P. capsici*, three cDNA libraries representing mycelia grown in V8 liquid medium (MY), swimming zoospores (ZO) and germinating cysts with germ tubes (GC) were constructed. We used a cellophane membrane placed on the host leaf as a surface for the induction of cyst germination in order to carry out molecular investigation of the early infection events by *P. capsici* but without the involvement of plant biomass into later processing. The cysts germinating on both cellophane membrane (Figure 1A) and tobacco leaf (Figure 1B, 1C) were highly similar, demonstrating the germination processes on two different surfaces were nearly synchronous and therefore the validity of this system for inducing the cyst germination. We did not harvest the germinating cysts in water because without the induction of surface martrix or host-derived compounds, the cyst germination was slower and not uniform, and only about 30% cysts were germinated in water after 90 min incubation (Figure 1D). Hence, RNA from the cysts germinating on cellophane membrane was extracted at 70 min post inoculation and used for Illumina RNA-Seq together with the MY and ZO RNA samples.

Each sequenced sample yielded 100-bp reads from single-end sequencing of cDNA fragments. After stringent quality assessment and data clearance, 11 to 12 million (M) reads with ∼94% Q20 bases (those with a base quality greater than 20) were selected as high quality reads for each library and used in the later analysis (Table 1). An average ‘G+C’ content of above 50% (51.01%, 55.18%, 54.55% for MY, ZO, GC libraries, respectively) was observed for the *P. capsici* ESTs. The high quality reads produced in this study have been deposited in the NCBI Sequence Read Archive database, accessible through the accession number SRP024305.

**Table 1:** Description of three *Phytophthora capsici* RNA-Seq libraries.

Library name	Cycle number	Total reads	Total bases	G+C content (%)	Cycle Q20 (% )
MY	100	11,047,203	1,104,720,300	51.01	94.67
ZO	100	11,696,377	1,169,637,700	55.18	95.87
GC	100	12,066,318	1,206,631,800	54.55	94.41

MY, vegetative growth mycelia; ZO, zoospores; GC, germinating cysts with germ tubes.

### Sequence mapping to the *P. capsici* reference genome

The distinct reads were compared against the *P. capsici* LT1534 genome that contains 19,805 gene models. The results showed that a substantial proportion of reads of each library (88.39% in MY library, 83.78% in ZO library and 80.60% in GC library) were matched to *P. capsici* genes (Table 2). Of them, the majority of mapped reads were ‘Perfect mapped reads’ without one base pair mismatch or Indel (48.47% in MY library, 45.44% in ZO library and 44.86% in GC library). After mapping reads to the gene models of *P. capsici* reference genome, 13,901, 14,633 and 14,695 putative genes were identified from the reads of the MY, ZO and GC libraries, respectively (Material S1). These data indicated that approximately 70% of *P. capsici* genes predicted in reference genome were expressed during the pre-infection stages, with more transcripts present in the GC stage than in the other two stages.

**Table 2:** Summary of Illumina reads mapping to *Phytophthora capsici* reference genome by BLAT analysis.

Library name	Total reads	Mapped reads^a^	Perfect Mapped reads^b^	Mismatched Reads^b^	Indel reads^b^	Indel and mismatched reads^b^	Identities^c^
MY	11,047,203	9,764,549	4,732,924	2,414,413	1,991,724	625,488	99.41%
	100%	88.39%	48.47%	24.73%	20.40%	6.41%	
ZO	11,696,377	9,799,624	4,453,292	4,565,083	410,353	370,896	98.94%
	100%	83.78%	45.44%	46.58%	4.19%	3.78%	
GC	12,066,318	9,725,254	4,362,412	4,125,646	810,886	426,310	99.01%
	100%	80.60%	44.86%	42.42%	8.34%	4.38%	

MY, vegetative growth mycelia; ZO, zoospores; GC, germinating cysts with germ tubes.

a The percentages below numbers for each library indicate the ratios of ‘Mapped reads’ to ‘Total reads’.

b The percentages below numbers in separate columns indicate the ratios of mapped reads in each case stated to ‘Mapped reads’.

c The percentages under this term for each library indicate the ratio of the number of mapped nucleotides without mismatching to that of total nucleotides.

Sequencing depth of three libraries was determined by saturation analysis. The saturation analysis was performed to check whether the number of detected genes keep increasing when the total amount of sequencing reads increases. Figure S2 showed a similar trend of saturation for three libraries where the number of detected genes almost ceases to increase when the number of reads reaches 10 M or higher. This indicates that three libraries were sequenced to saturation, generating a full representation of the transcripts in the conditions tested.

**Figure 2:**
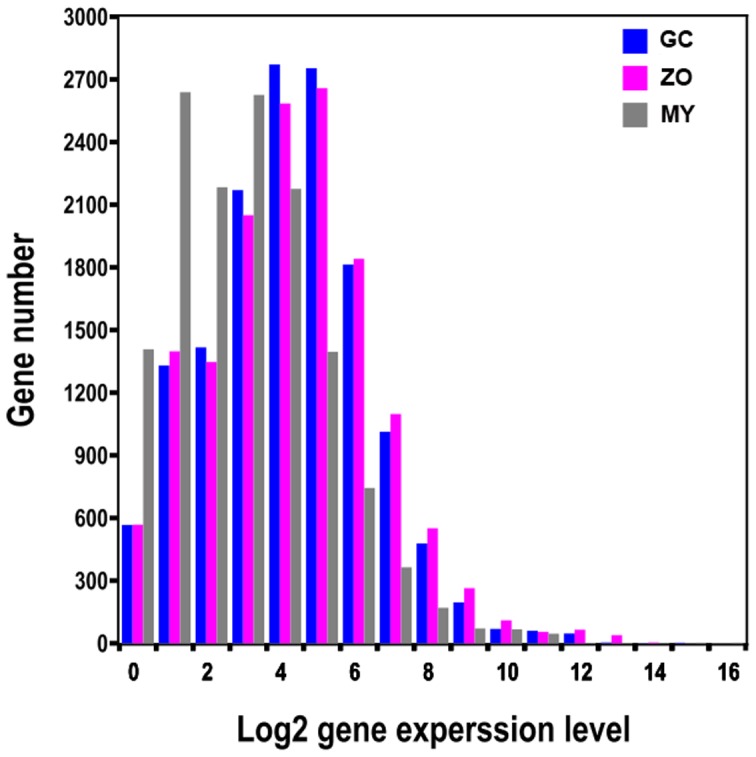
The distribution of gene expression levels. Total reads from each of three libraries that match to the 19,805 *P. capsici* gene models were plotted as integrated log_2_ values. The distribution is based on the number of genes falling in each log_2_ gene expression category.

Heterogeneity and redundancy are two significant characteristics of mRNA expression. Therefore, the distribution of gene expression levels was used to evaluate the normality of the library data. The level of gene expression was determined by calculating the number of reads for each gene and then normalizing this to the RPKM. As shown in Figure 2, the majority of mRNA is expressed at low levels whereas a small proportion of mRNA is highly expressed. As for the GC, the highest number of genes (2,753) distributes around 4 (≥4 and <5, and 4 refers to 16 RPKM) and 0.78% of the genes (115 of 14,695) are highly expressed at more than 10. The distribution showed the similar patterns for all three libraries.

### Gene expression during the pre-infection stages

**Figure 3:**
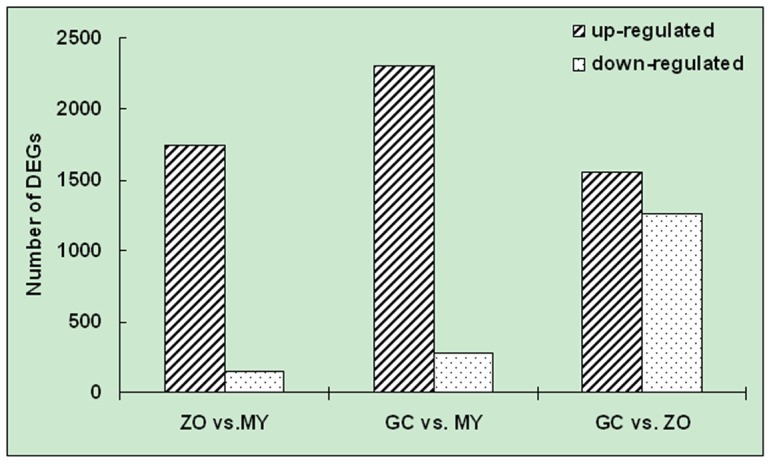
Changes in gene expression profile among the different developmental stages. The number of up-regulated and down-regulated genes between library pairs is summarized.

**Figure 4:**
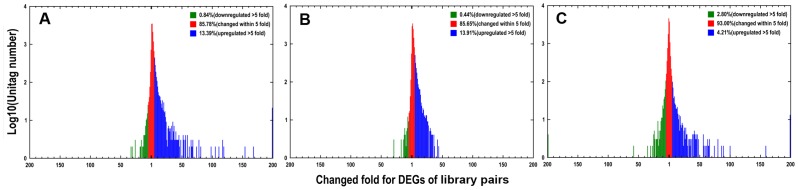
Differentially expressed genes in different stages. The ‘x’ axis represents fold-change of differentially expressed unique reads in different library pairs. The ‘y’ axis represents the number of unique reads (log_10_). Differentially accumulating unique reads within 5-fold difference between pairs are shown in the red region. The blue and green regions represent unique reads that are up- and down-regulated more than 5 fold in the stages, respectively. Library pairs: (A), GC vs. MY; (B) ZO vs. MY; (C) GC vs. ZO.

We analyzed the gene expression variations during the pre-infection stages of *P. capsici*. To study the differentially expressed genes between each library pair (GC-MY, ZO-MY and GC-ZO), we performed filtering using IDEG6 program to identify twofold up-regulated and twofold down-regulated genes with *P* value < 0.01, employing General Chi-squared test and FDR correction. The results, shown in Figure 3, indicate that the greatest changes in gene expression occurred during cyst germination (GC compared with MY and ZO). Between GC and MY libraries, a total of 2,578 DEGs were detected with 2,302 up-regulated and 276 down-regulated genes (Material S2). Between ZO and MY libraries, there were 1,898 DEGs detected with 1,742 up-regulated and 155 down-regulated genes (Material S2). By contrast to ZO, at GC there were more differentially expressed genes when compared with MY. This also confirmed that the GC was a distinct group. For GC–ZO pair, the gene-expression pattern changed steadily but not dramatically (1,552 up-regulated and 1,265 down-regulated genes) (Material S2; Figure 3). The DEGs with five-fold or greater differences in accumulation were shown in Figure 4. The distribution of genes corresponding to different fold change categories showed that the expression of 7.01–14.35% of genes changed by at least five fold while the expression level of the majority of genes was within fivefold difference.

The *P. capsici* genome sequence is publicly available. However, the annotation of Phyca11 gene models has not been completed. To get annotations for DEGs of three library pairs, we mapped them to additional 8 databases (NCBI Nr and Nt databases, Swiss-Prot, TrEMBL, COG, GO, KEGG and *P. infestans* genome databases). Altogether, the comparisons against these databases achieved annotations for most of the DEGs (1,893 of 1,898 for ZO-MY, 2,567 of 2,578 for GC-MY and 2,803 of 2,817 for GC-ZO) (Material S3).

A total of 98 *P. capsici* effector genes with known or putative roles in virulence [reviewed by 16, 17, 63] for library pairs of samples were identified (Table 3). These genes were grouped into classes defined as RXLRs, CRNs, elicitins, transglutaminase elicitors, NLPs, CBELs (cellulose binding elicitor lectins) and enzyme inhibitors (Table 3; Material S4). Most of the genes in these families were differentially expressed with a significant *P* value (*P* < 0.01) in GC versus MY and ZO. Of the RXLRs, 20 showed increased expression at GC, whereas four decreased, compared with MY. Of the CRNs, three increased at GC, whereas no genes decreased, compared with MY. Surprisingly, all 13 CRNs showed increased expression at ZO, compared with MY (Table 3).

**Table 3:** The effector genes differentially expressed during the pre-infection stages of *Phytophthora capsici.*

		Differentially expressed genes^b^								
		Pair GC - MY			Pair ZO - MY			Pair GC - ZO		
Gene category	Total^a^	GC	MY	No change	ZO	MY	No change	GC	ZO	No change
RXLR	32	20	4	8	7	0	25	22	5	5
Crinker (CRN)	13	3	0	10	13	0	0	0	2	11
Elicitin	24	13	7	4	9	2	13	9	10	5
Transglutaminase elicitor	7	4	0	3	1	3	3	5	0	2
NLP	8	3	0	5	0	0	8	7	0	1
CBEL	4	3	0	1	0	0	4	2	1	1
Enzyme inhibitor	10	7	1	2	3	0	7	2	3	5
Total	98	53	12	33	33	5	60	47	21	30

MY, vegetative growth mycelia; ZO, zoospores; GC, germinating cysts with germ tubes. Crinker (CRN), crinking and necrosis-inducing protein; NLP, NPP1-like protein; CBEL, cellulose binding elicitor lectin.

a Total number of *P. capsici* effector genes, homologous to *P. infestans* genes.

b Genes had a FDR < 0.05 and *P* < 0.01.

### Pathway enrichment analysis of DEGs

KEGG pathway enrichment analysis was performed to categorize the biological functions of DEGs. We mapped all the genes to terms in KEGG database. Specific enrichment of genes was observed for 264 pathways in the comparison of the GC and the MY, including 229, 35 pathways affected by up- and down-regulated DEGs, respectively (Material S5). A total of 208, 33 pathways were affected by up- and down-regulated DEGs, respectively, between the pair ZO vs. MY (Material S5). In contrast, a total of 155, 162 pathways were affected by up- and down-regulated DEGs, respectively, between the pair GC vs. ZO (Material S5). The majority of the DEGs were significantly up-regulated in the GC and ZO. The first ten enriched pathways between library pairs were reported in Table 4. Notably, ribosomal-associated proteins constituted the top pathway affected by the up-regulated DEGs among the three library pairs. The other pathways for up-regulated DEGs are mainly involved in secondary metabolism (such as purine metabolism, pyrimidine metabolism and glycolysis), energy metabolism, splicing, and protein synthesis. There were enriched pathways affected by the down-regulated DEGs, which were also involved in the secondary metabolism and energy conversion. However, up-regulated DEGs involved in the pathways were more than down-regulated DEGs. These results agree with the findings from the DEG analysis and suggest that the metabolism and signal transduction pathways might be more active in the GC and ZO.

**Table 4:** List of first ten pathways for up- and down-regulated DEGs between library pairs.

Pathway term	Pathway ID	DEGs tested	*P* value
**Library GC vs. MY**			
**Pathways for up-regulated DEGs**			
Ribosome	ko03011	59	3.63e-14
Ribosome biogenesis in eukaryotes	ko03008	44	1.35e-10
Purine metabolism	ko00230	40	2.15e-04
Translation factors	ko03012	33	1.38e-08
Chaperones and folding catalysts	ko03110	31	8.17e-03
RNA transport	ko03013	28	0.3723
Energy production and conversion	ko05016	26	0.0770
Spliceosome	ko03041	25	0.9999
Pyrimidine metabolism	ko00240	24	0.0074
Chromosome	ko03036	21	0.9999
**Pathways for down-regulated DEGs**			
Peptidases	ko01002	4	0.9999
Lipid biosynthesis proteins	ko01004	4	0.4758
Glycolysis / Gluconeogenesis	ko00010	3	0.0888
Biosynthesis of unsaturated fatty acids	ko01040	3	0.0466
Nitrogen metabolism	ko00910	3	0.0263
Lysosome	ko04142	3	0.9003
Lipid transport and metabolism	ko03320	2	0.0269
Arginine and proline metabolism	ko00330	2	0.0924
Chaperones and folding catalysts	ko03110	2	0.0082
Oxidative phosphorylation	ko00190	2	0.9915
**Library ZO vs. MY**			
**Pathways for up-regulated DEGs**			
Ribosome	ko03011	82	4.43e-42
Energy production and conversion	ko05016	38	2.75e-07
Oxidative phosphorylation	ko00190	28	7.35e-07
Mitochondrial electron transport	ko05010	26	1.91e-06
Energy metabolism	ko05012	25	1.09e-07
Ubiquitin system	ko04121	24	0.8363
Spliceosome	ko03041	24	0.9994
GTP-binding proteins	ko04031	23	1.12e-12
Peptidases	ko01002	19	0.9892
DNA repair and recombination proteins	ko03400	18	0.9823
**Pathways for down-regulated DEGs**			
Amino acid related enzymes	ko01007	5	0.9776
Amino acid transport and metabolism	ko00710	3	0.4536
Propanoate metabolism	ko00640	3	0.7245
Alanine, aspartate and glutamate metabolism	ko00250	3	0.6568
Valine, leucine and isoleucine biosynthesis	ko00290	2	0.7885
Phenylalanine, tyrosine and tryptophan biosynthesis	ko00400	2	0.6987
Glycine, serine and threonine metabolism	ko00260	2	0.9539
Cysteine and methionine metabolism	ko00270	2	0.7632
Butanoate metabolism	ko00650	2	0.1886
Oxidative phosphorylation	ko00190	2	7.35e-07
**Library GC vs. ZO**			
**Pathways for up-regulated DEGs**			
Ribosome biogenesis in eukaryotes	ko03008	39	6.31e-09
Purine metabolism	ko00230	20	0.3194
Chaperones and folding catalysts	ko03110	19	0.0102
Protein processing in endoplasmic reticulum	ko04141	15	0.1253
Nitrogen metabolism	ko00910	15	0.0001
Amino acid related enzymes	ko01007	14	0.2509
Spliceosome	ko03041	12	0.9976
Glycolysis / Gluconeogenesis	ko00010	11	0.0255
Alanine, aspartate and glutamate metabolism	ko00250	11	0.0357
Proteasome	ko03051	10	3.31e-05
**Pathways for down-regulated DEGs**			
Ribosome	ko03011	28	0.1461
Peptidases	ko01002	27	0.3993
Ubiquitin system	ko04121	20	0.9881
Energy production and conversion	ko05016	18	0.4984
Proteasome	ko03051	18	3.31e-05
Oxidative phosphorylation	ko00190	18	0.0780
GTP-binding proteins	ko04031	16	6.05e-05
Spliceosome	ko03041	16	0.9976
Mitochondrial electron transport	ko05010	16	0.1487
Energy metabolism	ko05012	15	0.0818

MY, vegetative growth mycelia; ZO, zoospores; GC, germinating cysts with germ tubes.

### Expression analysis by RT-PCR

Nineteen genes representing the families described above (8 RXLRs, 2 CRNs, 6 elicitins and 3 NLPs) were selected for confirmation as well as to monitor their expression during the host infection with RT-PCR (Table S1). Hereafter, we refer to them by their JGI protein designations, as that is what they are related with, with ‘Pc’ (for *P. capsici*) at the beginning of each name.

**Figure 5:**
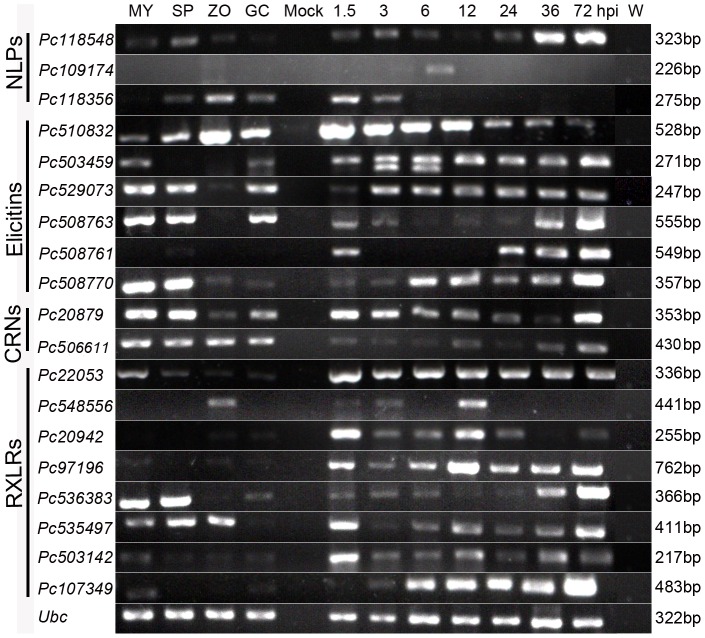
Expression patterns of 19 *Phytophthora capsici* genes during pre-infection and infection stages. Lanes: MY, plate-grown mycelia; SP, sporangia; ZO, zoospores; GC, germinating cysts; Mock, mock-inoculated plants; 1.5 – 72 hpi, *Nicotiana benthamiana* roots at 1.5, 3, 6, 12, 24, 36, 72 h post-inoculation (hpi); W, sterile distilled water. Gene names under different categories are listed on the left and sizes are listed on the right. All sizes were as expected, and the experiment was performed with at least three biological replicates.

All the genes were examined for expression in four developmental stages (mycelia, sporangia, zoospores and germinating cysts) and in *N. benthamiana* root samples at seven time-points after inoculation (1.5, 3, 6, 12, 24, 36 and 72 hpi) as well (Figure 5). The setup of these inoculation time-points assures that the whole infection period of *P. capsici* in *N. benthamiana* could be investigated. The disease progressed quickly in the plants, and by 5 dpi all inoculated plants were collapsed. As shown in Figure S1, *N. benthamiana* root rot was evident at 36 hpi. Brown lesions appeared in the middle of the roots and stems (Figure S1), but no evident macroscopic symptoms were observed on the leaves until 72 hpi. No symptoms developed in the control plants.

Consistent with the RNA-Seq results, the majority of the genes (15/19) were expressed during at least one of the pre-infection stages. The remaining four genes (*Pc109174*, *Pc508761*, *Pc20942* and *Pc97196*) showed low expression during these pre-infection stages. However, all tested genes showed increased expression in infection stages compared with developmental stages. Noticeably, most RXLR genes were expressed most highly at early infection time points, except *Pc536383* and *Pc107349*, which showed highly expression at later infection stages (Figure 5).

### *P. capsici* elicitin and NLP effectors cause cell death in *N. benthamiana*

**Figure 6:**
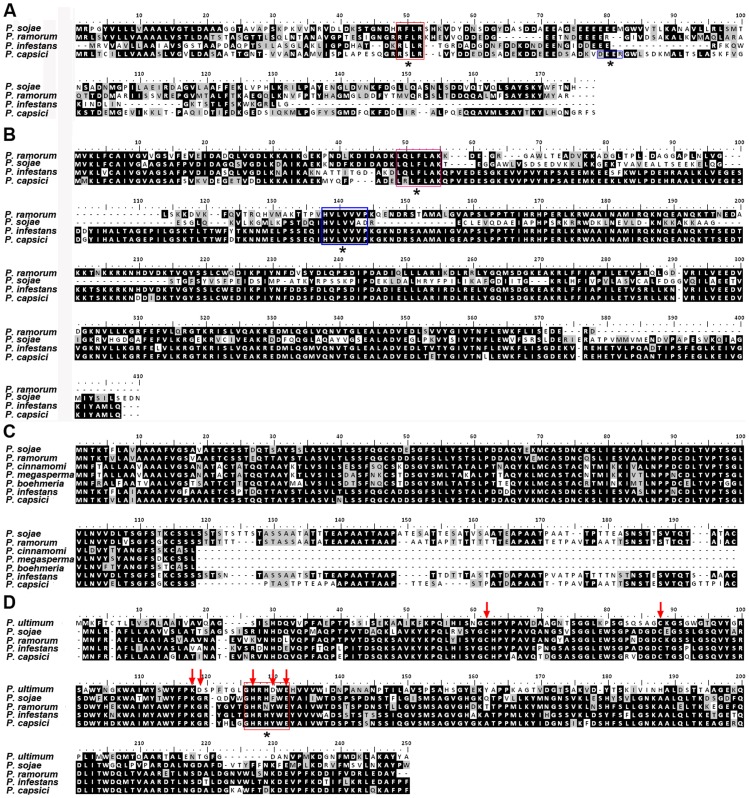
Alignment of four protein sequences from *Phytophthora capsici* against different *Phytophthora* species and *Pythium ultimum*. Full-length *P. capsici* homologous sequences were translated using BioEdit. (A) Pc22053. Red and blue boxes denote putative RXLR and dEER motifs, respectively. (B) Pc506611. Red and blue boxes denote putative LXLFLAK and HVLVVVP motifs, respectively. (C) Pc508761; (D) Pc118548. Red arrows indicate conserved cysteines and key functional residues previously described [24]. The putative GHRHDWE motif was denoted by red box. All motifs mentioned above were also marked by black asterisks for visualization.

Expression studies indicated that we had identified potentially important infection-related genes in *P. capsici*, including RXLRs, CRNs, elicitins and NLPs. To determine functionality, initially we cloned and performed multiple sequence alignment of four effector genes: *Pc22053* (RXLR), *Pc506611* (CRN), *Pc508761* (elicitin) and *Pc118548* (NLP). Full-length copies of selected genes were obtained by designing primers based on the *P. capsici* LT1534 gene models, and cloned into TA vector pMD-18T. The SignalP hidden Markov model (HMM) probability values of the putative proteins deduced from the selected genes were higher than 0.98. For SignalP NN mean S score, all the effectors had a value higher than 0.700, except Pc506611 (0.584) (Table 5). The resulting translated amino acid sequences were used for alignments (Figure 6). A single species was chosen to represent each of five *Phytophthora* clades [64]. BLAST results with the predicted proteins showed that *P. capsici* Pc22053, Pc506611, Pc508761 and Pc118548 have 18%, 91%, 50% and 78% identity to *P. infestans* homologues, respectively (Table 5). The open reading frame (ORF) of RXLR gene *Pc22053* is 519-bp long without any introns, which was mis-annotated as containing a 120-bp intron in the reference genome. The predicted protein showed 15 amino acids including RXLR-dEER motif conserved across three different *Phytophthora* species whereas 32 amino acids were shared with *P. infestans*. The CRN effector Pc506611 shared 100 amino acids including the conserved LXLFLAK and HVLVVVP motifs with three different *Phytophthora* species, whereas 368 of 402 amino acids (91%) were conserved between both *P. capsici* and *P. infestans*. The elicitin Pc508761 revealed that, of 182 amino acids in *P. capsici*, 54 were conserved across six different *Phytophthora* species. Conversely, 92 amino acids were conserved when aligned with only *P. infestans*. One NLP effector of interest, Pc118548, showed 86 of 246 amino acids conserved across three different *Phytophthora* species and an additional oomycete, *Pythium ultimum*, whereas 193 were shared with *P. infestans*, corresponding to 78% identity. The data showed that all aligned NLPs have two conserved cysteine residues, which define these proteins as type I NLP [23], [65]. We also noted that the central conserved GHRHDWE heptapeptide motif is present in all NLPs, but has changed in sequence in NLPs from the selected *Phytophthora* species but not from *Py. ultimum* (Figure 6).

**Table 5:** Summary and comparison of putative RXLR, CRN, elicitin and NLP protein sequences from *Phytophthora capsici* with *Phytophthora infestans.*

			Gene	SignalP	SignalP	SignalP	% ID^c^	Number of amino acids (total)	Number of amino acids (conserved)^d^	
Gene	GenBank accession no.	Genome position^a^	length (bp)	HMM^b^	NN^b^	length^b^	*P. capsici*		*P. infestans*	
*Pc22053*	KF220580	scaffold_535:1187–1705	519	1.000	0.906	22	18	172	102	32
*Pc506611*	KF220579	scaffold_20:748014–749251	1209	0.988	0.584	17	91	402	404	368
*Pc508761*	KF220578	scaffold_38:51106–51767	549	1.000	0.771	20	50	182	188	92

^a^ Genome position refers to the above genes’ position in the *P. capsici* genome sequence assembly database v11.0.

b Hidden Markov model (HMM) probability, NN mean S score, and signal peptide length were predicted using SignalP v3.0.

c Blasted against the *P. infestans* genome sequence.

d Conserved between both *P. capsici* and *P. infestans*.

**Figure 7:**
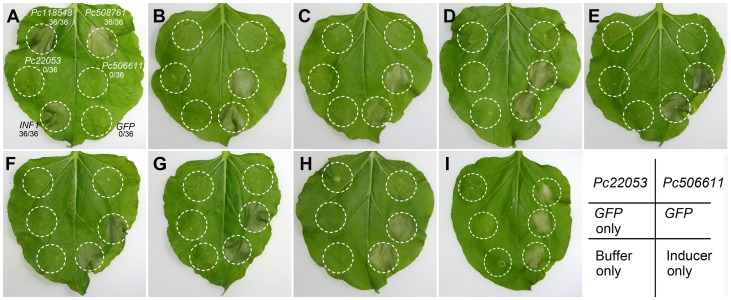
Transient assays of *Phytophthora capsici* RXLR, CRN, NLP and elicitin effectors in *Nicotiana benthamiana*. (A) Pc118548 (NLP) and Pc508761 (elicitin) trigger cell death in *N. benthamiana*. The plant leaves were infiltrated with *A. tumefaciens* (strain GV3101) cells to express *Pc22053* (RXLR), *Pc506611* (CRN), *Ps118548*, *Pc508761*, *P. infestans INF1* (positive control) or *GFP* (negative control). Photographs were taken 5 d after infiltration. (B) – (I), Pc22053 and Pc506611 suppress cell death caused by different cell death inducers in *N. benthamiana*. Agro-infiltration sites in each *N. benthamiana* leaf expressing *Pc22053* (top left), *Pc506611* (top right), or *GFP* (middle right) were challenged with *A. tumefaciens* expressing *Bax* (B), *INF1* (C), *Pc508761* (D), *PsojNIP* (E), *Pc118548* (F), *PsAvh241* (G), *PsCRN63* (H) and *Avr3a*/*R3a* (I). The other three sites in each leaf expressing *GFP* only (middle left), inducer itself (bottom right) or infiltrated by 10 mM MgCl_2_ buffer only (bottom left) served as controls. Photographs were taken 5 d after cell death inducer infiltration.

The selected genes then were sub-cloned into the PVX vector pGR107. Transient expression assays with these four genes were performed on *N. benthamiana* plants. We tested whether they could individually induce necrosis in *N. benthamiana* using infiltration of *A. tumefaciens* cells with pGR107 carrying each of the genes. These methods have been widely used for transient expression of genes in *N. benthamiana* and for determining whether particular genes can induce or suppress cell death [18], [32], [34], [37], [59], [66]–[68]. The known elicitor of cell death, *P. infestans INF1*, was used as a positive control. Agro-infiltration of *INF1*, *Pc508761* and *Pc118548* induced hypersensitive response (HR)-like host cell death, as indicated by the macroscopic necrosis, whereas only mosaic symptoms due to PVX were observed following agro-infiltration of *Pc22053*, *Pc506611* or *GFP* under the same conditions (Figure 7A). *Pc508761* induced an HR within 3 days like *INF1* whereas *Pc118548* triggered an HR at 4 days post-infiltration.

### *P. capsici* RXLR and CRN effectors suppress host cell death

To further explore the functions of *Pc22053* (RXLR) and *Pc506611* (CRN), we tested whether they could suppress cell death like other bacterial and oomycete effectors [32], [37], [59], [66], [69]. *Pc508761*, *Pc118548* plus six additional cell death inducers were used in this assay: *BAX*, *INF1*, *PsojNIP*, a combination of *Avr3a* and *R3a*, *Avh241* and *PsCRN63*. Cell death symptoms were observed when *A. tumefaciens* cells carrying the above genes were infiltrated into *N. benthamiana* leaves (Figure 7B-7I). However, when *Pc22053* was infiltrated in *N. benthamiana* leaves 24 h prior to infiltration of each of the inducers, this pretreatment blocked cell death triggered by all 8 cell death inducers. By comparison, *Pc506611* could protect *N. benthamiana* tissue from cell death triggered by all tested cell death inducers but *R3a*/*Avr3a* (Figure 7I). Furthermore, prior infiltration with *A. tumefaciens* cells containing the *GFP* gene, a negative control, did not protect against cell death (Figure 7B-7I).

## Discussion

We present a deep transcriptional study of the oomycete *P. capsici*, a pathogen of interest because of its damage to diverse crops and its genetic relatedness to other important oomycete pathogens [14]. We anchored the *P. capsici* transcriptome using its draft genome as a reference [70], investigated the global gene expression in three important life cycle stages (MY, ZO and GC) and identified a large number of genes associated with specific stages and pathogenicity. We identified 13,901, 14,633 and 14,695 genes from the reads of the MY, ZO and GC libraries, respectively (Material S1). We showed that about 70% of the *P. capsici* genes in LT1534 genome are represented in our study. The remaining 30% of conserved eukaryotic genes may not have been sampled because not all developmental and infection stages of *P. capsici* were represented in our material (e.g. sporangium, but also sexual reproduction).

Comparative analysis between two of samples showed major differences between the expressed gene content of MY, ZO and GC stages. A suite of genes associated with specific stages and pathogenicity were identified between two of samples (Material S2 and S3). These transcriptional shifts generally agreed with the biological process of the pathogen. This was confirmed by the analysis of differentially expressed genes, which showed major transitional shifts between the libraries from the different groups. The distinction of ZO and GC from MY is probably due to its divergence from non-mycelium status (e.g., they are either swimming in water or attached to the host surface to germinate). The GC was an especially distinct group with more differentially expressed genes detected, probably because the initial host-pathogen interaction occurred during this stage can reasonably be expected to modify the pathogen’s expression profile. Additionally the GC stage is transitory between the ZO and MY stages. The results of KEGG pathway enrichment analysis (Table 4; Material S5) lend support to the biological significance of gene expression profiles derived from the deep sequencing, which will assist in the discovery and annotation of *P. capsici* genes playing key roles in development and particularly in pre-infection stages. It is not surprising that the ‘ribosome-related’ pathways were the most affected for the DEGs more common in ZO and GC libraries. This finding implies that the oomycete utilizes new ribosomes or changes in ribosome components to help synthesize additional proteins to facilitate its swimming towards potential host plants and subsequent germination on host surface.

Sequencing of plant pathogenic oomycetes has revealed a variable number of effectors in different species. In the present study, 99 *P. capsici* effector genes were identified with known or putative roles in virulence (Table 3; Material S4). We predicted that these RXLRs, CRNs, NLPs and elicitins would be highly expressed during pre-infection and infection stages. Our results partially supported our hypothesis, in that these effectors were mostly induced *in planta* (Figure 5). A study by Haas et al. [18], in which the authors examined genome-wide expression in *P. infestans* over four time points from 2 to 5 dpi of potato, revealed similar results that in general RXLRs showed early expression. Interestingly, the elicitin class showed the induction at GC and infection stages for *P. capsici*, whereas there were no elicitins induced at all during infection by *P. infestans*. DEG analysis showed that in contrast to ZO (13), only 3 CRNs showed increased expression at GC compared with MY. This implied that some CRNs were probably repressed at GC stage (an important pre-infection stage). In a recent study on *P. capsici* CRNs, based on contrasting gene expression profiles, Stam et al. [36] defined two classes of CRN effectors (Class 1 and 2). We noted from the RT-PCR result that two tested CRN genes fell into Class 1 featuring high levels of expression at the early time points, a decrease during subsequent biotrophic stages and expression in the later stages (Figure 5). However, another similar study by Kunjeti et al. [68] showed *Phytophthora phaseoli* CRNs were mostly repressed *in planta*. A transcriptional study on *P. sojae* showed induction of RXLRs, NLPs, CRNs and elicitins was high at the GC stage and another peak later in infection stage on soybean [71]. These data suggest that *P. capsici* expresses some genes in common with other *Phytophthora* pathogens, but the timing may differ.

Our RXLR dataset includes several predicted orthologs to known avirulence genes such as *P. sojae Avr1b* and *P. infestans Avr3a* [62], [72]. For instance, the predicted protein Pc22053 showed 33% identity with *P. sojae* Avr1b (BLASTx, *E*-value = 2e-05). However, *Pc22053* can not cause cell death in *N. benthamiana* during the transient expression assays (Figure 7A). This is not surprising as data from *P. sojae* indicate that *Avr1b* induced cell death occurs only on soybean plants carrying *Rps1b* [73]. To explore this further, we tested for transcripts of *Pc22053* by RT-PCR and observed its highly expression during the host infection (Figure 5). Furthermore, we observed that Pc22053 can suppress the HR induced by all 8 different effectors (Figure 7B-7I). Similarly, selected RXLR effectors and the RXLR-like SNE1 protein from other *Phytophthora* species have demonstrated their ability to suppress cell death and defense [27], [29], [32], [59]. We demonstrated that one *P. capsici* CRN gene *Pc506611* does not cause plant cell death when transiently expressed in *N. benthamiana* (Figure 7A). This is not a rare case because recent studies show that cell death induction is not a universal feature of CRN proteins. Over-expression of *Phytophthora* CRN domains only induce necrosis in a few cases [18], [36], [37]. Intriguingly, when the putative Pc506611 CRN protein sequence was compared against NCBI Nr database, a ubiquitin-like (UBL) domain was detected in this protein (Position 13–58; BLASTP, *E*-value = 3.99e-03). The family of proteins containing UBL and ubiquitin-associated domains has been implicated in proteasomal degradation [74]. Whether the protein contributed to the pathogenicity through ubiquitin pathway during the host-oomycete interaction is yet to be determined. Furthermore, the high expression of *Pc506611* during the GC and early infection stages (Figure 5) leads us to suspect that it contributes positively to the pathogen’s virulence. Ectopic expression showed that this gene can suppress plant cell death caused by all tested cell death inducers but *R3a*/*Avr3a* (Figure 7B-7I). Data from a recent study on *P. sojae* CRNs indicate that one CRN, PsCRN115, also suppresses cell death elicited by e.g. *PsojNIP* and *PsCRN63* [37]. The similar results indicated that this family of effectors also has similar abilities to RXLR effectors in suppressing plant defense. Host cell death or the HR is an effective and ultimate defense mechanism against obligate biotrophic and hemibiotrophic pathogens [75]. However, delayed HR could be counterproductive and benefit hemibiotrophic pathogens in necrotrophic growth stages. *P. capsici* is a hemibiotroph and switches from biotrophic to necrotrophic growth 18 to 42 h following the invasion of *N. benthamiana* leaves [14]. Therefore, the ability of *P. capsici* to suppress or delay the HR of host tissue is likely a major component of its pathogenic strategy, as is for *P. sojae* [59], [76].

We observed a small number of NLPs and elicitins with differential expression (Table 3; Material S4; Figure 5). DEG analysis showed that three NLPs showed increased expression at GC compared with MY. RT-PCR analysis demonstrated that they are highly expressed during the infection stages. Similarly, a study on *P. sojae* NLPs found that most NLP are highly expressed during cyst germination and infection stages [67]. Ectopic expression showed that one of our *P. capsici* NLP genes (*Pc118548*) triggered cell death in *N. benthamiana* (Figure 7A). Although it is accepted that NLPs can contribute to virulence as toxins, it has been reported that many members of this family do not possess obvious toxic activity [24], [67]. The expansion of NLP gene families in *Phytophthora* spp. has been noted in whole-genome analyses and the size of the NLP family in *P. infestans* (n  = 27), *P. sojae* (n  =  39), and *Phytophthora ramorum* (n  =  59) has been estimated through annotation [17], [18]. Recently, 18 putative NLP genes have been cloned from *P. capsici* strain SD33 [77]. However, the exact number of functional or ‘real’ genes encoding NLPs in *P. capsici* is yet to be determined and their exact functional roles in *P. capsici* virulence remain to be elucidated.

In total 24 elicitin genes were detected in the present study by Illumina sequencing. DEG analysis showed that 13 and 9 elicitin genes were up-regulated at GC and ZO stages compared with MY, respectively (Table 3; Material S4). Gene expression analyses showed that *P. capsici* elicitin genes were mostly induced during the host infection (Figure 5). A study on infection-related gene changes in *P. phaseoli* by Kunjeti et al. [68] revealed similar results. They observed that the expression of four elicitin genes was increased while that of 2 decreased. Ye et al. [71] also reported the induction of elicitins was high at the germinated-cyst stage and infection stage on soybean. Transient assay showed that one of *P. capsici* elicitin genes (*Pc508761*) induced plant cell death (Figure 7A). Future studies will involve an exploration of whether these elicitins are required for host infection or sterol acquisition [78].

## Conclusions

*P. capsici* is a widespread and destructive plant pathogen and in particular presents an attractive model for understanding oomycetes with broad-host-ranges. Here, we have demonstrated that deep sequencing of the transcriptome combined with computational tools such as DEG profiling has provided a wealth of information about the genes differentially expressed during the pre-infection stages of *P. capsici*. Our findings point to potentially important *P. capsici* RXLR and CRN effectors that can modulate host defense circuitry and benefit parasite colonization. An important challenge in the next step is to determine how these effector proteins manipulate host processes. This dataset will ultimately help to elucidate the mechanisms underlying the pathogenicity and to design novel control strategies for the diseases caused by *P. capsici*.

## Supporting Information

Figure S1 **The disease progressions in** ***Nicotiana benthamiana*** **roots caused by** ***Phytophthora capsici*** **isolate Pc537.** Each plant root was cut open before macroscopic observations. Arrows indicate the inoculation part or browning, necrosis and rot symptoms. (A) controls (mock-inoculated); (B-D) the plants at 24, 36, 72 h post-inoculation, respectively.
(TIF)

Figure S2 **Accumulation of Illumina total reads and unique genes in the three libraries.** New unique genes (‘y’ axis) of MY, ZO, GC libraries (different color lines) decreased as the depth of sequencing (‘x’ axis) increased.
(TIF)

Table S1 **List of primers used for RT-PCR and cloning of the** ***Phytophthora capsici*** **genes.**
(XLS)

Material S1 ***Phytophthora capsici*** **genes expressed during mycelium, zoospore and germinating cyst stages.** (listed in separate sheets for each stage).
(XLS)

Material S2 **Differentially expressed genes between two samples.** (3 sample-pairs listed in separate sheets).
(XLS)

Material S3 **Differentially expressed genes between two samples with annotation.** (3 sample-pairs listed in separate sheets).
(XLS)

Material S4 **The effector genes differentially expressed during the pre-infection stages of** ***Phytophthora capsici.*** (7 different categories listed in separate sheets with annotation).
(XLS)

Material S5 **Complete list of involved pathways for regulated DEGs between two samples.** (3 sample-pairs listed in separate sheets).
(XLS)
