# Correction: RNA-Seq Reveals Infection-Related Gene Expression Changes in *Phytophthora capsici*

**DOI:** 10.1371/annotation/3be2cab2-9116-49c1-acce-7d51f4ccd887

**Published:** 2013-09-13

**Authors:** Xiao-Ren Chen, Yu-Ping Xing, Yan-Peng Li, Yun-Hui Tong, Jing-You Xu

There was an error in Table 5. A correct version of the error in available here: http://www.plosone.org/corrections/pone.0074588.g005.tif

