# Supplementary figures and images for: RNA-Seq Reveals Infection-Related Gene Expression Changes in Phytophthora capsici
Source: PLoS One. 2013 Sep 3;8(9):e74588. doi: 10.1371/journal.pone.0074588 (PMC3760852; doi:10.1371/journal.pone.0074588)

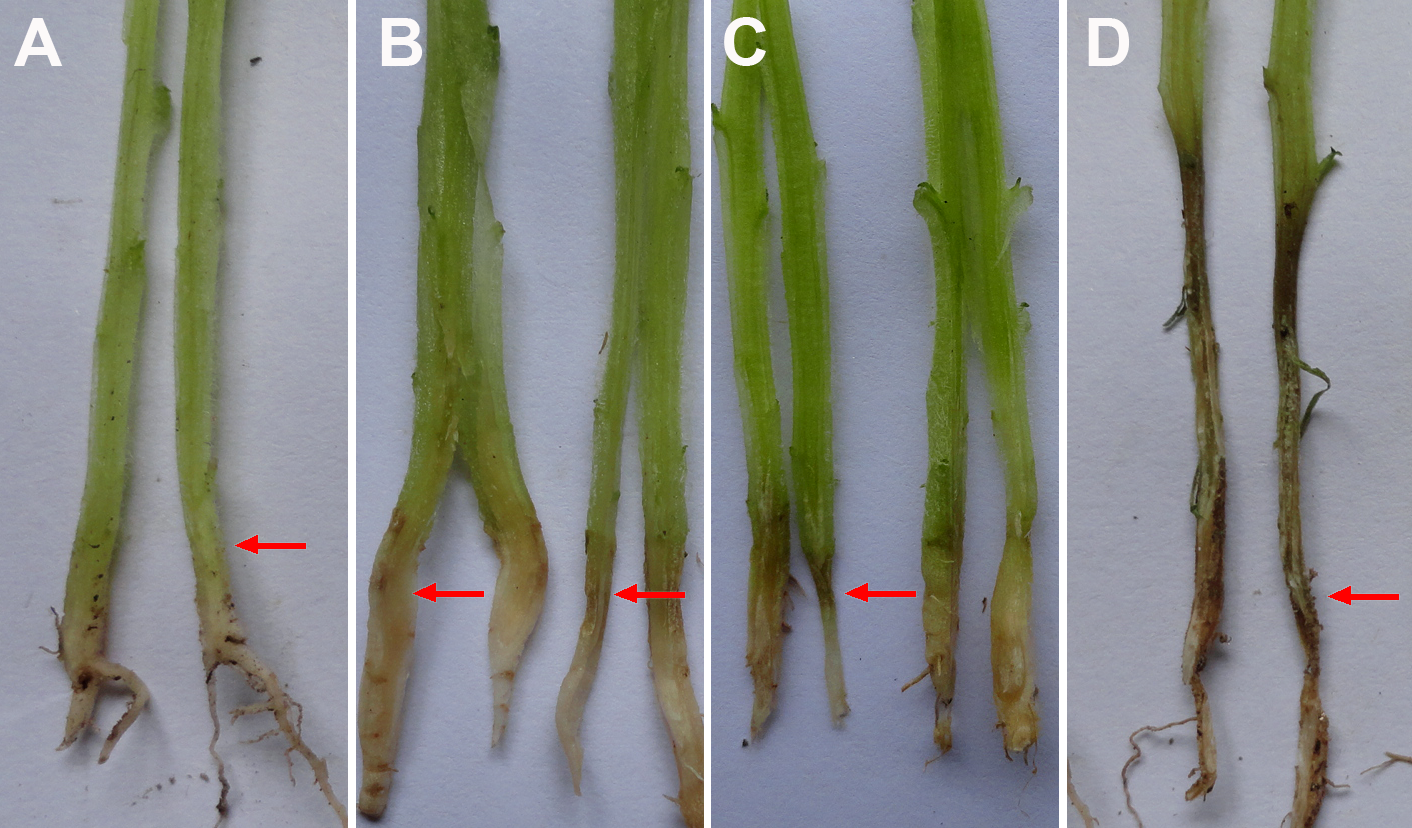

Supplement: Figure S1 — The disease progressions in Nicotiana benthamiana roots caused by Phytophthora capsici isolate Pc537. Each plant root was cut open before macroscopic observations. Arrows indicate the inoculation part or browning, necrosis and rot symptoms. (A) controls (mock-inoculated); (B-D) the plants at 24, 36, 72 h post-inoculation, respectively. (TIF) [file pone.0074588.s001.tif]

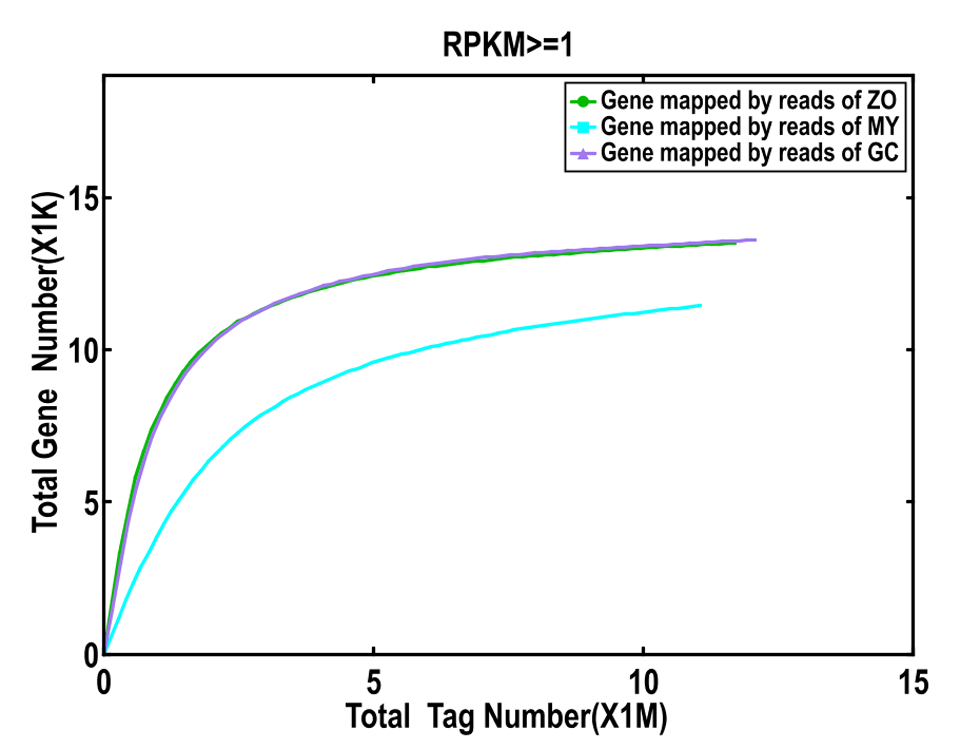

Supplement: Figure S2 — Accumulation of Illumina total reads and unique genes in the three libraries. New unique genes (‘y’ axis) of MY, ZO, GC libraries (different color lines) decreased as the depth of sequencing (‘x’ axis) increased. (TIF) [file pone.0074588.s002.tif]
